# Serum Neurotrophin Profile in Systemic Sclerosis

**DOI:** 10.1371/journal.pone.0013918

**Published:** 2010-11-09

**Authors:** Marie-Claude Lise, Agnès Sparsa, Isabelle Marie, Fabrice Lalloué, Kim Ly, Clothilde Martel, Holy Bezanahary, Guillaume Gondran, Véronique Loustaud-Ratti, Jean-Marie Bonnetblanc, Elisabeth Vidal, Marie-Odile Jauberteau, Anne-Laure Fauchais

**Affiliations:** 1 EA3842 Homéostasie Cellulaire et Pathologies, IFR 145, Limoges University Hospital, Limoges, France; 2 Department of Internal Medicine, Limoges University Hospital, Limoges, France; 3 Department of Dermatology, Limoges University Hospital, Limoges, France; 4 Department of Immunology, Limoges University Hospital, Limoges, France; 5 Department of Internal Medicine, Rouen University Hospital, Rouen, France; Institut Jacques Monod, France

## Abstract

**Background:**

Neurotrophins (NTs) are able to activate lymphocytes and fibroblasts; they can modulate angiogenesis and sympathic vascular function. Thus, they can be implicated in the three pathogenic processes of systemic sclerosis (SSc). The aims of this study are to determine blood levels of Nerve Growth Factor (NGF), Brain-Derived Neurotrophic Factor (BDNF) and Neurotrophin-3 (NT-3) in SSc and to correlate them with clinical and biological data.

**Methods:**

Serum samples were obtained from 55 SSc patients and 32 control subjects to measure NTs levels by ELISA and to determine their relationships with SSc profiles.

**Findings:**

Serum NGF levels were higher in SSc patients (288.26±170.34 pg/mL) than in control subjects (170.34±50.8 pg/mL, *p*<0.001) and correlated with gammaglobulins levels and the presence of both anti-cardiolipin and anti-Scl-70 antibodies (*p*<0.05). In contrast, BDNF levels were lower in SSc patients than in controls (1121.9±158.1 *vs* 1372.9±190.9 pg/mL, *p*<0.0001), especially in pulmonary arterial hypertension and diffuse SSc as compared to limited forms (all *p<*0.05). NT-3 levels were similar in SSc and in the control group (2657.2±2296 *vs* 2959.3±2555 pg/mL, NS). BDNF levels correlated negatively with increased NGF levels in the SSc group (and not in controls).

**Conclusion:**

Low BDNF serum levels were not previously documented in SSc, particularly in the diffuse SSc subset and in patients with pulmonary hypertension or anti-Scl-70 antibodies. The negative correlation between NGF and BDNF levels observed in SSc and not in healthy controls could be implicated in sympathic vascular dysfunction in SSc.

## Introduction

Systemic sclerosis (SSc) is a chronic connective tissue disease characterized by at least 3 pathogenic processes: immunological abnormalities, micro-vascular dysfunction and fibrosis [1].

Neurotrophins (NTs) belong to a family of growth factors that control the development, growth and apoptotic death of neurons and astrocytes [2]. Accumulating evidences suggest that NTs, especially Nerve Growth Factor (NGF) and Brain-Derived Neurotrophic Factor (BDNF), participate in inflammatory responses, including the modulation and regulation of immune functions in inflammatory and autoimmune diseases [2]. NGF serum levels are increased in various autoimmune diseases such as systemic lupus erythematosus and rheumatoid arthritis [3]. Increased NGF and BDNF plasmatic levels have also been recently reported in primary Sjögren's syndrome in correlation with systemic activity and B and T cell activation [4].

Neurotrophins could also be implicated in the generalized microangiopathy observed in SSc. Indeed, angiogenesis, endothelial cell activation, apoptosis and sympathetic vasoconstriction are modulated by these neuropeptides. NGF promotes angiogenesis and synthesis of angiogenic factors such as Vascular Endothelial Growth Factor (VEGF) [5]. As recently evidenced, BDNF can induce angiogenesis in ischemic tissues [6]. In acute coronary syndromes, ischemic tissues contain increased BDNF levels that correlate with inflammation and oxidative stress whereas serum levels are decreased [7]. Furthermore, pulmonary expression of p75^NTR^, the low affinity NT receptor, regulates endothelial susceptibility to endothelin-1 [8]. Thus, NTs could regulate agonist-induced pulmonary vasoconstriction [8].

NGF is able to induce both fibroblast proliferation and collagen production [9]. These pro-fibrogenic properties of NGF are mediated by transforming growth factor-beta (TGFβ) [10], [11], a key cytokine in the pathogenesis of SSc related fibrosis [12].

However, data concerning the implication of NTs in SSc are sparse and restricted to NGF. Skin NGF expression is increased in SSc patients compared to healthy controls, especially in the early stages of the disease [13]. The same group reported increased blood NGF levels in SSc, especially in the diffuse subset of the disease and in patients with prominent articular disease [14].

The aim of the present study was to evaluate serum levels of NGF, BDNF and NT-3 in patients with SSc and to investigate their relationship with clinical and immunological data.

## Materials and Methods

### Patients and control population

Fifty five consecutive SSc patients including 49 women (median age 54.2±12.5 years), all fulfilling the revised American College of Rheumatology (ACR) criteria for SSc were included in a cross sectional study in two French SSc competence centers by using the same screening protocol [15]. Mean disease duration at time of the study was 5.5±3.3 years. Disease stages were defined as suggested by Medsger and Steen: early limited SSc, disease duration <5 years; intermediate/late limited SSc, disease duration ≥ 5 years, early diffuse SSc, disease duration <3 years and intermediate/late diffuse SSc, disease duration ≥ 3 years [16].

Patients with evolutive neoplastic disorders or depression were excluded in order to avoid interferences in serum NTs levels [17].

The control population consisted of 32 age- and sex-matched healthy volunteers. Informed consent was obtained from all patients and control subjects who participated in the study, which was approved by the local ethic review board “Comité d'éthique de la commission d'établissement du CHU de Limoges” directed by Dr G Terrier (35-2009-17).

### Clinical feature

The disease was classified as diffuse (dSSc) or limited SSC (lSSc) according to the degree of skin involvement [18]. Four patients have anti-RNP Ab and presented with an overlap syndrome, with clinical features evoking lupus erythematosus in 3 cases and polymyositis in one case. The diagnoses of each disease component were determined according to ACR criteria.

A clinical profile was determined in every SSc patient (Table 1). Skin involvement was measured by the modified Rodnan skin score on a scale of 0-51 [19]. The presence of fingertip ulcers at the time of blood sampling and the existence of previous digital ulcers were systematically recorded. Lung disease was assessed by pulmonary function tests, annual X-rays (n = 55) and computed tomography scans (n = 11) performed within 12 month before blood sampling. For the interpretation of the pulmonary function tests, FVC (liters, percentage of predicted forced vital capacity), DLCO (CO ml/min/mmHg and percentage of predicted capacity of carbon monoxide), DLCO/alveolar volume values (percentage of predicted) and 6 minutes walk test (meters and percentage of predicted) were measured and compared to those obtained 6 to 12 months earlier. Pulmonary arterial hypertension (PAH) was defined by a mean pulmonary artery pressure >25 mm Hg at right heart catheterization. Joint involvement was recorded as positive when there was objective evidence of tender or swollen joints, or both. Disease severity was scored by the Medsger's index [20].

**Table 1:** Clinical, immunological profiles and functional respiratory tests characteristic of patients with systemic sclerosis (SSc).

**Clinical characteristics**			
Age, *y*	*(mean ± sd)*	54.5±12	
Female, *n(%)*		43 (93)	
Classification of scleroderma spectrum of disease, *n (%)*			
	Diffuse scleroderma	9 (19)	
	Limited scleroderma	33 (72)	
	Overlap syndrome	4 (9)	
Skin involvement, Rodnan score *(mean ± sd)*		9.3 ±7.5	
Digital ulcers, *n (%)*			
	evolutive	5 (11)	
	previous	15 (33)	
Articular involvement, *n (%)*		15 (33)	
Pulmonary fibrosis, *n (%)*		1 (2)	
Pumonary hypertension, *n (%)*		2 (4)	
	associated with pulmonary fibrosis	1 (2)	
	isolated	1 (2)	
Digestive complications, *n (%)*		18 (39)	
Associated Sjögren syndrome, *n (%)*		9 (19)	
**Functional respiratory tests characteristics**			
	FVC (*liters mean ± sd; % mean ± sd)*	3.45±0.89	110±16.2
	DLCO *(CO ml*/*min*/*mmHg mean ± sd*; *% of predicted*)	18.6±4.47	76.5±13.5
	DLCO/VA *(% of predicted*)		88.7±15.4
	6 month-previous DLCO/VA *(% of predicted*)		86.2±15.9
	6 minutes walk test *(meters mean ± sd; % of predicted*)	440.45±97.8	84.4±11.2
**Immunological characteristics**			
	Anti-nuclear Ab, *n (%)*	44 (96)	
	Anti-Scl70 Ab, *n (%)*	3 (7)	
	Anti-centromere Ab, *n (%)*	33 (71)	
	Anti-cardiolipin Ab, *n (%)*	8 (17)	

FVC: forced vital capacity, DLCO: diffusing lung factor for carbon monoxide, DLCO/VA diffusing capacity divided by the alveolar volume. sPAP: Systolic pulmonary artery pressure at right heart catheterism. Ab: antibodies.

Concomitant treatment of SSc patients included angiotensin-converting enzyme inhibitors (n = 14), calcium channel blockers (n = 43), endothelin inhibitors (n = 6), aspirin (n = 12) and statins (n = 12). Eight patients had cyclic intravenous iloprost infusions for severe Raynaud's phenomenon within the six-month period before blood sampling. Fifteen patients were receiving immunomodulating drugs at the time of blood sampling: low doses of corticosteroids (n = 10, mean 11±6.3 mg/day), hydroxychloroquine (n = 5), methotrexate (n = 5) cyclophosphamide (n = 2) and mycophenolate mofetil (n = 1).

### Measurement of autoantibodies gammaglobulins and neurotrophins levels

Anti-nuclear, anti-centromere and anti-Scl70 antibodies (Ab) were characterized by immunofluorescence on HEp2 cells (The Binding Site, Saint Egrève, France) and by ELISA (Phadia, Saint Quentin Yvelines, France). Anti-cardiolipin Ab were measured by ELISA (Ingen, Chilly Mazarin, France).

Total serum gammaglobulins were measured by serum protein immunoelectrophoresis.

Serum NGF, BDNF and NT3 levels were measured using commercial ELISA kits according to the manufacturer's instructions (NGF E_max_® ELISA, BDNF E_max_® ELISA, NT-3 ELISA, Promega, Charbonnières, France). All assays were performed in duplicate and the data are presented as pg/mL. Detection limits were 15 pg/mL for BDNF and 4 pg/mL for NT-3 and NGF. Coefficients of variability for the individual samples run in the ELISAs were 0.99, 0.94 and 1.17 for NGF, BDNF and NT3, respectively.

### Determination of BDNF production by B and T cells

Expression of intracellular BDNF by T and B lymphocytes was assessed by flow cytometry in 5 SSc patients and 5 controls. Whole blood cells were stained with either phycoerythrin (PE)-cyanin (Cy) 7-conjugated anti-CD3 or anti-CD19 antibodies (Ab) for 15 min at room temperature. After red blood cell lysing (Immunoprep, Beckman Coulter, France), white blood cells were fixed, permeabilized (Intraprep, Beckman Coulter, France) and incubated at room temperature for 30 min with mouse anti-BDNF Ab (1/100; Santa Cruz Biotechnology, France) in Phosphate Buffered Saline (PBS) containing 1% Bovine Serum Albumin. After two washes in PBS, mAb were revealed using Alexafluor 488-conjugated goat anti-mouse IgG Ab (10 µg/mL; Invitrogen, France) for 30 min at 4°C. Cells stained with mouse isotypic immunoglobulins (Santa Cruz Biotechnology, France) were used as controls to determine background and positivity thresholds. After washing twice in PBS, cells were suspended in PBS and analyzed with a flow cytometer (FacsCanto™ II).

### Statistical analysis

The results were expressed as means ± standard deviations. *p* values ≤0.05 were considered significant. One-way analysis of variance (ANOVA), chi-square tests and Mann-Whitney tests were used when appropriate. To search for correlations between serum NTs levels and clinical and other biological data, linear regression analysis was used and *p*-values determined by Spearman's rank correlation test.

## Results

### Variations in NTs expression in SSc

**Figure 1:**
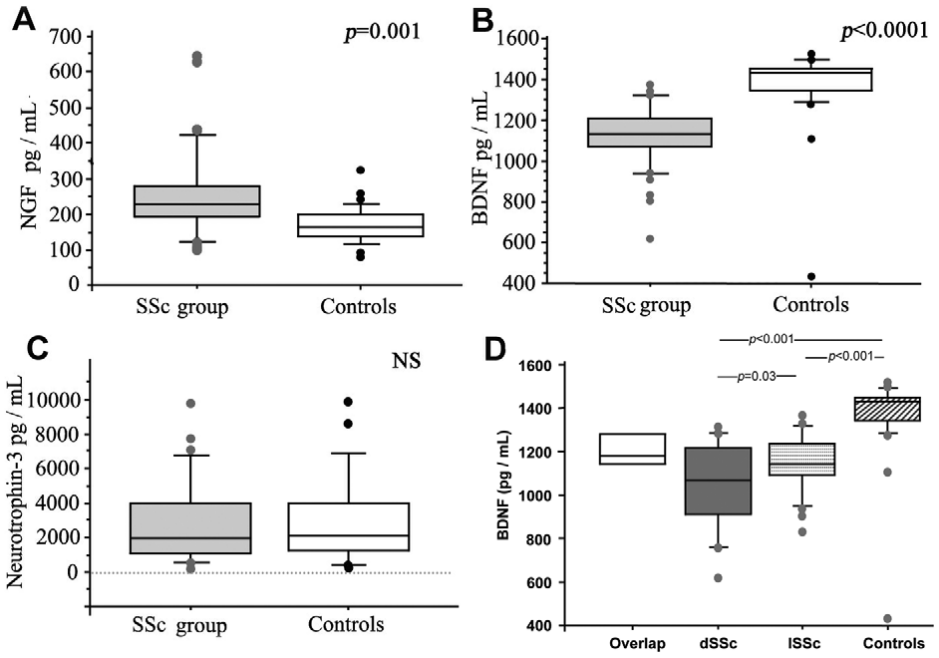
(A) Nerve Growth Factor (NGF), (B) Brain-Derived Neurotrophic Factor (BDNF) and (C) Neurotrophin-3 (NT-3) concentrations measured by ELISA in systemic sclerosis (SSc) patients compared to healthy controls (Controls) (Mann-Whitney tests). The boxes represent the 50^th^ percentiles while the bars outside the boxes show the 10^th^ and 90^th^ percentiles (•); the horizontal grey lines represent median values. (D) BDNF serum levels in overlap syndrome (white box), limited SSc (lSSc, grey box) and diffuse SSc (dSSc grey dotted box) (Mann-Whitney tests).

Serum NGF levels were higher in SSc patients (288.26±170.34 pg/mL) than in healthy controls (170.34±50.8 pg/mL, *p*<0.0001) (Figure 1A) whereas BDNF levels were lower (1121.9±158.1 *vs* 1372.9±190.9 pg/mL) (Figure 1B). Serum NT-3 levels were similar in SSc and in controls (2657.2±2296 *vs* 2959.3±2555 pg/mL, NS) (Figure 1C).

The lowest BDNF concentrations were those of patients affected with dSSc (1045.67±202.8 pg/mL), as compared with lSSc patients (1146±126.8 pg/mL, *p* = 0.03) (Figure 1D). In contrast, serum NGF levels were similar in patients with dSSc (346.16±210 pg/mL), lSSc (271.4±136.5 pg/mL) and overlap syndrome (195.5±52 pg/mL).

To examine whether the variation of NGF and BDNF is a feature of both early and intermediate/late stages of the disease, serum samples were analyzed according to disease duration. In all subgroups of patients defined by this criterion, levels of BDNF were significantly reduced than in healthy controls (BDNF early dSSc 1041±33.8 and late dSSc 1067.2±182 pg/mL, NS) and (BDNF early lSSc 1140.9±151.5 and late lSSc 1144.84±116.6, NS) vs controls (1372.9±190.9 pg/mL, all p<0.003).

NGF levels were also similar in early (204.9±53.4 pg/ml) and late dSSc (440.1±354.7 pg/mL, NS) as well as in lSSc (260±138.5 pg/mL) and late lSSc (249.8±101.1 pg/mL, NS).

### NGF and BDNF balance

**Figure 2:**
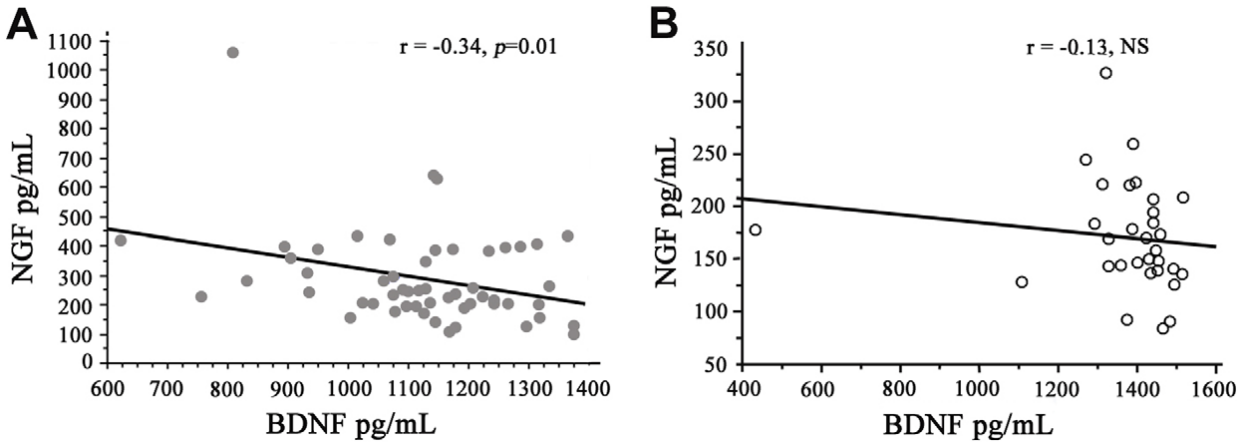
(A) Correlation between Brain-Derived Neurotrophic Factor (BDNF) and Nerve Growth Factor (NGF) levels in (A) SSc patients (grey circles) and (B) healthy controls (white circles). The linear regression curve and coefficient of determination given by the analysis of variance are both represented. Each point represents an individual patient and a healthy control. r defines the coefficient of determination given by the analysis of the variance table. *p*-values were determined by Spearman's rank correlation test.

A strong negative correlation was observed between reduced BDNF levels and increased NGF concentrations in the whole SSc group (r = −0.33, *p* = 0.01, Figure 2A), in contrast to the lack of any correlation between these levels in the control group (r = −0.13, NS, Figure 2B). No correlation could be observed between NT-3 serum levels and NGF and BDNF concentrations either in the SSc group (NGF r = 0.11, NS; BDNF r = 0.05, NS) or in the controls (NGF r = 0.06, NS; BDNF r = 0.09, NS).

### Neurotrophins and SSC clinical profile

Whereas BDNF levels were reduced in the subgroup of patients with PAH (922.8±235.6 *vs* 1135.8±145.4 pg/mL in the absence of PAH, *p* = 0.05); patients with grade II, III and IV PAH had similar BDNF decreased serum levels (893.9, 898 and 1130 pg/mL, respectively). In contrast, NGF concentrations were similar in patients with or without PAH (393.2±143.8 *vs* 293.6±169.8 pg/mL, NS).

BDNF levels were not significantly different in patients with (1072.4±95.7 pg/mL) and without (1128±163.8 pg/mL) fingertip ulcers (NS). Similarly, there was no correlation between NGF levels and the presence of fingertip ulcers.

The presence of articular involvement or digestive complications did not influence serum levels of NGF, BDNF and NT-3. NGF, NT-3 and BDNF concentrations were also similar in SSc patients with or without secondary Sjögren syndrome. No correlation of NGF, BDNF and NT-3 levels with the Rodnan skin score was found either.

BDNF levels were lower in patients with severe (according to Medsger's classification) SSc (1055.1±127.8 pg/mL) than in those with mild forms (stade 2, 1162.8±104 pg/mL, *p* = 0.05). In contrast, NGF and NT-3 levels were similar in all Medsger's classification groups.

**Figure 3:**
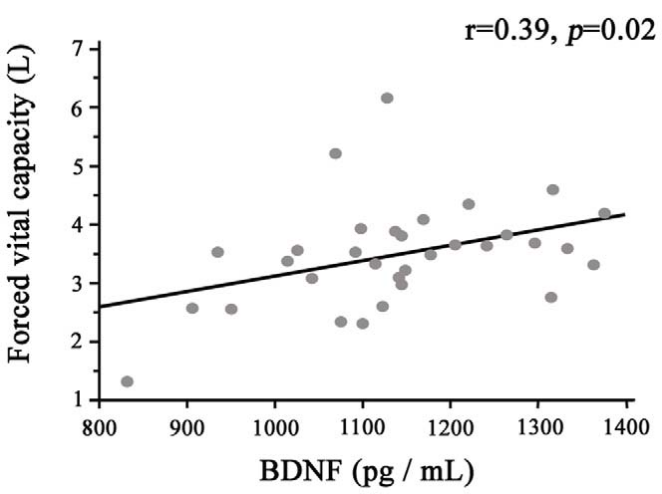
Correlation between Brain-Derived Neurotrophic Factor (BDNF) concentrations and forced vital capacity in limited SSc group (lSSc, grey circles). Each point represents an individual patient. r defines the coefficient of determination given by the analysis of variance table. *p*-value was determined by Spearman's rank correlation test.

A significant correlation between BDNF and FVC (r = 0.39, *p* = 0.02) (Figure 3) was found in the lSSc group. There were no significant correlation between serum NTs levels and carbon monoxide diffusion capacity, DLCO/VA or 6-minutes walk tests. Declines of DLCO/VA or 6-minutes tests observed during a 6 months follow-up failed to correlate with serum NTs levels also.

Serum NGF, BDNF and NT-3 concentrations were similar in SSc patients whatever they were treated or not by angiotensin-converting enzyme inhibitors, calcium channel blockers or statins. NGF serum concentrations tended to be slightly higher in patients receiving aspirin (347.9±246.1 pg/mL) than in those without platelet aggregation-inhibiting drug (261±122.7 pg/mL, p = 0.08, NS). NGF and BDNF levels were also similar in SSc patients with or without endothelin inhibitors. However in the subgroup of patients with active or previous digital ulcers, NGF levels were reduced in the 5 patients given endothelin inhibitor therapy (215.4±115.9 pg/mL) as compared to the 19 others (313.2±89 pg/mL, *p* = 0.04). Disease-modifying drugs, except hydroxychloroquine did not affect serum NTs levels; NT-3 levels were higher in SSc patients with than without (4555.6±3933.3 *vs* 2474.5±2031.6 pg/mL, *p* = 0.05) hydroxychloroquine, an immuno-modulative drug prescribed for severe articular manifestations in 80% of the cases.

### Neurotrophins and immunological profile

**Figure 4:**
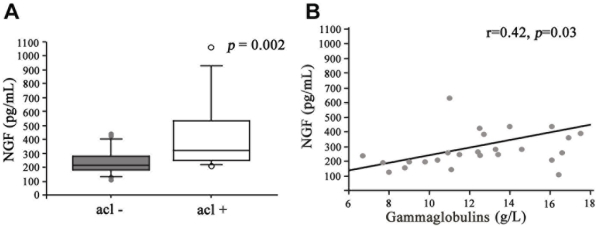
(A) Nerve Growth Factor (NGF) concentrations (ELISA) in patients with (acl+) or without (acl-) anti-cardiolipin Ab (Mann-Whitney test). The boxes represent the 50^th^ percentiles while the bars outside the boxes show the 10^th^ and 90^th^ percentiles (•); the horizontal grey lines represent median values. (B) Correlation between Nerve Growth Factor (NGF) concentrations and gammaglobulins levels. The linear regression curve and coefficient of determination given by the analysis of variance are both represented. *p*-value was determined by Spearman's rank correlation test.

Patients with anti-Scl-70 antibodies showed significantly high NGF levels (408.3±241.4 *vs* 262±122.2 pg/mL, *p* = 0.008). NGF levels were also increased in patients with anti-cardiolipin antibodies (408.5±262 *vs* 264.2±99.2 pg/mL, *p* = 0.008) (Figure 4A) and correlated positively with serum gammaglobulin levels (r = 0.42, *p* = 0.03) (Figure 4B).

### BDNF production by B and T cells

Intracellular production of BDNF by B and T lymphocytes estimated by flow cytometry was similar in SSc patients and controls (mean fluorescence intensity of B cells 3.2±1.6 *vs* 2.3±1.1, NS and T cells 3.7±0.8 *vs* 2.9±1.5, NS).

## Discussion

The present study documents the yet unreported finding of reduced serum BDNF levels in SSc, particularly in the diffuse SSc subset, as well as in patients with severe forms or pulmonary hypertension. We confirm the increase of serum NGF concentrations previously reported in SSc [14]. Interestingly, NGF and BDNF levels correlated negatively in SSc patients and not in healthy controls. Although NT-3 has been recently shown to be involved in cardiovascular diseases [21], the present data fail to document its implication in SSc

The present results confirmed previous reports [13], [14], [22] that circulating levels of NGF are increased in SSc. The persistence of high serum NGF levels in intermediate-late forms of SSc strongly suggests of an implication of NGF in disease pathogeny independently of the disease duration. Although enhanced dermal levels of NGF reflect skin involvement in SSc, previous data and the present study suggest that increased serum NGF levels do not reflect skin or pulmonary fibrosis in SSc [14]. Indeed, no statistical correlation between the Rodnan score and serum NGF levels could be found. Whereas NGF production in the skin could reflect local inflammatory processes and fibrosis, we hypothesize that enhanced serum levels are a consequence of the autoimmune aspect of SSc, especially B cell chronic activation. Indeed, NGF levels are increased in SSc as well as in various others autoimmune disease such as lupus erythematosus, rheumatoid arthritis and primary Sjögren's syndrome [3], [4], [23]–[25]. NGF, produced by activated B and T cells, is involved in the maintenance of immunological memory B cells and it can provide a CD40-independent signal for immunoglobulin production [26], [27]. We herein evidence a statistical association between increased NGF levels and anti-Scl-70 and anti-cardiolipin Ab. We also confirmed the correlation between serum NGF concentrations and gammaglobulins levels, as previously demonstrated in primary Sjögren's syndrome [4]. Altogether, theses findings suggest an involvement of NGF in the B cell chronic activation in SSc.

Immunomodulating drugs did not affect NGF and BDNF levels in SSc. However, data concerning the influence of corticosteroid and/or immunosuppressive drugs on systemic levels of NGF and BDNF in autoimmune diseases are contradictory. Whereas serum NGF levels can be down-regulated by corticosteroid in asthma [28], immunomodulating drugs do not appear to reduce NGF levels in rheumatoid arthritis or primary Sjögren's's syndrome [4], [29]. The same discrepancy was reported for BDNF in the same diseases [28], [30]. Although pro-inflammatory cytokines play a role in promoting NT secretion, other mechanisms are probably involved in NGF and BDNF release in autoimmune diseases. High serum NT-3 levels in patients receiving hydroxychloroquine are intriguing. No previous report deals with NT-3 modulation by anti-malarial drugs.

Low BDNF levels were not previously reported in SSc. They are likely unrelated to the immune component of SSc. Indeed, serum BDNF levels have been reported to be increased in autoimmune diseases such as rheumatoid arthritis and primary Sjögren's syndrome independently of immunosuppressive treatment [4], [29]. No previous reports deal with serum BDNF levels in skin fibrosis; accordingly, BDNF serum levels did not correlate with Rodnan skin score in the present study. Therefore, we hypothesize that decreased BDNF levels do not reflect skin fibrosis in SSc.

Serum BDNF concentrations reflected his production by various cell types including both endothelial cells [31] and circulating lymphocytes [4], [32]. The production of BDNF by normal B and T lymphocytes is also modulated by cell activation [4], [33]. Local production of BDNF as well as the expression of its high affinity receptor TrkB is also enhanced in synovitis in autoimmune disease [34], [35]. However, BDNF production by B and T lymphocytes was similar in SSc patients and controls. Therefore, decreased BDNF concentrations are unlikely to reflect a direct modulation of lymphocytic BDNF production in SSc.

The decreased BDNF levels in SSc patients could be related to microvascular disease and oxidative stress. BDNF and his receptor TrkB are expressed by endothelial cells and vascular smooth cells of capillaries and arteries [36], [37]. A reduced BDNF synthesis by endothelial cells and the binding of serum BDNF to activated endothelial cells that express the tropomyosin receptor kinase B (TrkB) might play a role [38]. BDNF is implicated in vascular development and enhances the survival of vascular endothelial cells [37]. It enhances vascular flow and regulates revascularization of ischemic tissues also [39]. *In vitro* effects of BDNF on endothelial cells are comparable to those observed with VEGF [39]. Decreased BDNF concentrations have been observed in acute coronary syndrome [7], [40]. In addition, increased oxidative stress decrease serum BDNF concentrations [41], [42]. The positive correlation between serum BDNF levels and forced vital capacity could also reflect the potential link between this NT and oxidative stress. Forced vital capacity is known to decrease with enhanced oxidative stress in SSc [43], [44]. Furthermore, BDNF-induced activation of TrkB signaling *in vivo* promotes prostacyclin biosynthesis by cerebral arteries [45]. Altogether, decreased BDNF levels observed in SSc could reflect vascular damages. This hypothesis may be supported by the fact that the lowest levels of BDNF in this study were observed in the 5 patients with PAH.

The present paper reports on the intriguing finding that decreased BDNF and increased NGF levels correlate both in early and intermediate/late forms of SSc, which suggests a potential physiopathological role of this dysbalance. Such a serum NGF/BDNF balance is not observed normally nor in psychological stress, depression, autoimmune diseases or acute coronary syndrome [4], [7], [29], [46]. This BDNF/NGF balance abnormality observed in SSc patients might be a marker of chronic vascular dysfunction and oxidative stress. It is clear that NTs play an important role in maintaining normal vascular tone, oxidative homeostasis and in the regulation of the response of both endothelial and vascular smooth muscle cells to injury [35], [37]. Exogenous BDNF co-infused with NGF-receptor blocking Ab induces a marked vasoconstriction and perivascular inflammation [47], in accordance with the fact that NGF exerts a protective function in vascular control whereas BDNF alone has no significant effect on vascular diameter [47]. NGF reduces oxidative stress-induced injury by up-regulating antioxidants and oxygen free radical scavengers such as catalase, glutathione peroxidase and superoxide dismutase [48]. Hence, NGF can protect against post-ischemic dysfunction of sympathetic innervations and ischemia-reperfusion myocardial injury [49], [50]. In the present study, the relationship between oxidative stress and enhanced NGF levels could be deduced from the statistical relationship between NGF levels and anti-cardiolipin antibodies, which reflect vascular damage in SSc [51], [52]. The normalization of NGF levels in the 5 patients undergoing endothelin inhibitor therapy reinforces the assumption of the relationship between NGF levels and oxidative stress in SSc [53].

Altogether, reduced serum BDNF levels associated with vascular lesions and oxidative stress in SSc could be counterbalanced by “protective” increased NGF levels. Further studies and animal models should help to further elucidate the pathological relevance of NGF/BDNF balance, as well as uncover the precise mechanism of this interaction in the multifaceted pathogenesis of SSc.
